# Thrombosis in a bleeding disorder: case of thromboembolism in factor VII deficiency

**DOI:** 10.1002/ccr3.836

**Published:** 2017-02-01

**Authors:** Sheryl K. Ramdass, Kah Poh Loh, Leslie M. Howard

**Affiliations:** ^1^ Department of MedicineBaystate Medical CenterSpringfieldMassachusettsUSA; ^2^ Division of Hematology/OncologyJames P. Wilmot Cancer InstituteUniversity of Rochester/Strong Memorial HospitalRochesterNew YorkUSA; ^3^ Department of Hematology and OncologyBaystate Medical CenterSpringfieldMassachusettsUSA

**Keywords:** Anticoagulation, factor VII deficiency, pulmonary embolism, thrombosis

## Abstract

Congenital factor VII deficiency (FVIID) is a rare disorder with a wide range of bleeding manifestations. The disorder does not protect patients against occurrence of thrombosis, and deep vein thrombosis can occur in the setting of surgery and recombinant factor VIIa replacement.

## Introduction

Congenital factor VII deficiency (FVIID), the most frequent among the rare inherited bleeding disorders, is a heterogeneous disease with clinical manifestations ranging from asymptomatic to life‐threatening hemorrhage 1, 2. Paradoxically, FVIID does not protect affected patients from venous thromboembolism (VTE). We report a case of thrombosis in a patient with severe FVIID in the setting of surgery and recombinant factor VIIa.

## Case Presentation

A 73‐year‐old male presented following an episode of chest tightness, dyspnea, and presyncope 1 month after a right femoral neck fracture requiring internal fixation. During initial evaluation, acute coronary syndrome was ruled out. CT angiogram of the chest demonstrated multiple bilateral filling defects involving the lobar, segmental, and subsegmental pulmonary arteries of all lobes, consistent with pulmonary emboli. Doppler ultrasounds of the lower extremities revealed bilateral deep venous thrombosis. His past medical history was significant for factor VII deficiency (FVIID), diagnosed at an outside facility at age 44, following incidentally noted abnormal coagulation testing. He had never experienced significant bleeding episodes. There was no family history of bleeding disorders or venous thromboembolism. Previously, he had received single prophylactic doses of recombinant factor VIIa (rFVIIa) prior to cataract surgery and dental extraction with no bleeding complications. Laboratory testing prior to his recent hip surgery was significant for PT 27.8 sec (ref. range, 9.7–12.2 sec), INR 2.7, PTT 25.9 sec (ref. range, 24.1–33.1 sec), and factor VII activity level <5.0% (ref. range, 50–200%); no inhibitor was present (Table 1). Notably he did receive recombinant factor VIIa 20 *μ*g/kg immediately prior to surgery as well as every 8 h postoperatively for a total of four doses. PT corrected to 8.8 sec, INR 0.9, and factor VII activity level >200% (Table 1). There was minimal blood loss intraoperatively and no postoperative bleeding. He did not receive postoperative thromboembolism prophylactic anticoagulation. Following the diagnosis of venous thromboembolism during this current admission, he was started on standard doses of intravenous unfractionated heparin (UFH) overnight and then switched to subcutaneous low‐molecular‐weight heparin (LMWH) 1 U/kg twice daily the following day. UFH was initially chosen due to its short half‐life. His anti‐factor‐Xa level measured four hours following administration of LMWH was 0.91 IU/mL (ref. range, 0.6–1.1 IU/mL). The patient completed 3 months of anticoagulation in the context of provoked thromboembolism without any bleeding complications on LMWH. Unfortunately, he re‐presented 2 months following completion of anticoagulation with shortness of breath and was found to have a new left lower extremity deep venous thrombosis (DVT) and extensive bilateral pulmonary emboli. He was tested for factor V Leiden and factor II mutations; however, these returned negative. He was anticoagulated with UFH and transitioned to apixaban on discharge. The patient presented again 4** **months following that discharge with shortness of breath, generalized weakness, and fever secondary to *E. Coli* bacteremia likely genitourinary in origin. Notably the patient did admit to only taking apixaban for few days following previous discharge and self‐discontinued this medication due to diarrhea; he did not inform any medical providers. A CT angiogram of the chest showed overall decreased clot burden compared to previous studies, and Doppler ultrasounds of the lower extremities revealed chronic stable nonocclusive DVTs. Therapeutic anticoagulation was not restarted during this encounter given repeat imaging studies and timeframe. Two days later, he died of a cardiac arrest. No autopsy was performed.

**Table 1:** Coagulation profile and factor VII activity levels before and after administration of recombinant factor VIIa (rFVIIa) in the setting of hip surgery

	Prothrombin time, PT/sec (ref. range 9.7–12.2 sec)	International normalized ratio, INR	Activated partial thromboplastic time, APTT/sec (ref. range 24.1–33.1 sec)	Factor VII activity level (ref. range 50–200%)
Before rFVIIa	27.8	2.7	25.9	<5%
After rFVIIa	8.8	0.9	30.6	>200%

No inhibitor present.

## Discussion

The reported prevalence of FVIID is approximately one symptomatic individual per 500,000 population 3, but the true prevalence is likely higher due to a large proportion of asymptomatic or minimally symptomatic individuals. Inherited FVIID is an autosomal recessive disorder caused by a broad spectrum of mutations in the FVII gene located on chromosome 13. Factor VIIa initiates coagulation through the intrinsic pathway by binding to tissue factor exposed by vascular injury. FVIID is the only inherited bleeding disorder characterized by prolongation of the prothrombin time (PT), which corrects with administration of plasma, and a normal activated partial thromboplastic time (APTT).

There is a poor correlation between FVII coagulation activity (FVII:C) and bleeding tendency 4, 5, leading to difficulty in predicting bleeding risk**,** and hence, specific guidelines on preoperative management are lacking. This is exemplified by our patient whose baseline FVII: C level was <5%, but he had no prior significant history of bleeding. Paradoxically, thrombotic events can occur, as hemostatic defects do not seem to offer protection from strong thrombosis risk factors. Thrombotic episodes, particularly DVT, have been reported in 3–4% of patients with FVIID, even in severely deficient patients 4, 6. Although spontaneous thrombosis can occur, the majority of events have been reported to occur in the context of intensive replacement therapy, mostly rFVIIa or derived FXI concentrate, in the setting of surgical procedures 7, 8. Our patient had both of these risk factors; he underwent orthopedic surgery as well as received activated FVII preoperatively.

The literature on anticoagulation (AC) strategies for FVIID individuals who develop thrombosis is not well established. The tendency for both bleeding and thrombosis makes it challenging to find a safe and effective anticoagulant. The baseline INR is elevated in patients with FVIID; therefore, monitoring may be difficult with vitamin K antagonist agents. A small case series proposed that oral vitamin K antagonist would provide suboptimal AC as it preferentially decreases FVII levels in relation to the remaining vitamin K‐dependent factors, increasing bleeding risk in previously asymptomatic patients 9. There is also evidence that reduced factor II levels and not FVII provides antithrombotic protection in those receiving vitamin K antagonists 10. This strategy was initially considered in our patient; using Coumadin and monitoring factor II levels, however, due to potential increased bleeding risk and long laboratory assay time for factor II especially in the outpatient setting, this was not performed. Heparin, either unfractionated or LMWH, is also reported to be relatively safe in this population with monitoring anti‐Xa activity as an indicator of optimal antithrombotic therapy in cases where LMWH is used 4. Our patient did complete 3 months of LMWH therapy for his initial episode of thromboembolism without any reported bleeding episodes.

In conclusion, the clinician must be aware that thrombosis can occur in FVIID under certain provoked circumstances and severe deficiency does not offer protection against thrombosis. Venous thromboembolism prophylaxis should be considered in patients with predisposing risk factors for thrombosis including major surgery especially in those with no history of bleeding. The use of replacement products prior to surgery should also be individualized. Furthermore, our case highlights that therapeutic AC in the setting of thrombosis in patients with FVIID without a prior significant bleeding history can be safe, although future studies on the choice of AC is warranted especially in the era of novel oral anticoagulants.

## Authorship

SKR and LH: were involved in the clinical care of the patient. SKR: did the literature review and drafted the manuscript. KPL and LH: critically reviewed the manuscript for important intellectual content.

## Conflict of Interest

The authors have no conflict of interest or funding to disclose.
